# Genome-wide identification of soybean microRNA responsive to soybean cyst nematodes infection by deep sequencing

**DOI:** 10.1186/s12864-017-3963-4

**Published:** 2017-08-02

**Authors:** Bin Tian, Shichen Wang, Timothy C. Todd, Charles D. Johnson, Guiliang Tang, Harold N. Trick

**Affiliations:** 1 Department of Plant Pathology, Kansas State University, 1712 Claflin Road, 4024 Throckmorton Plant Sciences Center, Manhattan, KS 66506 USA; 2 Genomics and Bioinformatics Service, Texas A&M AgriLife, College Station, TX 77845 USA; 3 Department of Biological Sciences, Michigan Technological University, Dow Environmental Sciences and Engineering Building - Room 406, 1400 Townsend Drive, Houghton, MI 49931-1295 USA

**Keywords:** microRNAs, Soybean cyst nematode, Soybean, RNA-seq

## Abstract

**Background:**

The soybean cyst nematode (SCN), *Heterodera glycines*, is one of the most devastating diseases limiting soybean production worldwide. It is known that small RNAs, including microRNAs (miRNAs) and small interfering RNAs (siRNAs), play important roles in regulating plant growth and development, defense against pathogens, and responses to environmental changes.

**Results:**

In order to understand the role of soybean miRNAs during SCN infection, we analyzed 24 small RNA libraries including three biological replicates from two soybean cultivars (SCN susceptible KS4607, and SCN HG Type 7 resistant KS4313N) that were grown under SCN-infested and -noninfested soil at two different time points (SCN feeding establishment and egg production). In total, 537 known and 70 putative novel miRNAs in soybean were identified from a total of 0.3 billion reads (average about 13.5 million reads for each sample) with the programs of Bowtie and miRDeep2 mapper. Differential expression analyses were carried out using edgeR to identify miRNAs involved in the soybean-SCN interaction. Comparative analysis of miRNA profiling indicated a total of 60 miRNAs belonging to 25 families that might be specifically related to cultivar responses to SCN. Quantitative RT-PCR validated similar miRNA interaction patterns as sequencing results.

**Conclusion:**

These findings suggest that miRNAs are likely to play key roles in soybean response to SCN. The present work could provide a framework for miRNA functional identification and the development of novel approaches for improving soybean SCN resistance in future studies.

**Electronic supplementary material:**

The online version of this article (doi:10.1186/s12864-017-3963-4) contains supplementary material, which is available to authorized users.

## Background

MicroRNAs (miRNAs), a class of genome-encoded, endogenous small non-coding RNAs, play important roles in regulating gene expression at the transcriptional and post-transcription level by degrading corresponding mRNA or inhibiting its translation in plants [1, 2]. Plant miRNAs are known to regulate diverse biological processes, including plant growth and development, genome integrity, epigenetics, immunity against pathogens, and responses to environmental changes [3–7]. Thousands of miRNAs have been discovered from various plant species and new ones continue to be discovered. Coupled with new developments in high-throughput sequencing technology and gene expression profiles, research interests in the functional analysis of miRNAs are growing [8]. Although most of miRNAs are involved in controlling plant developmental and productive processes [9], studies in the past few years have demonstrated that miRNAs also play important roles in responses to infection by a variety of parasites including bacteria, fungi, oomycetes and nematodes [10–14]. Furthermore, both bacteria and *Phytophthora* pathogens produce virulent proteins that can suppress small RNA biogenesis for the benefit of infection [15, 16]. These findings suggest that miRNAs are integral regulators of plant defense and may have a conserved function in plant defense against various pathogens.

The soybean cyst nematode (SCN), *Heterodera glycines* (Ichinohe), is one of the most economically devastating pathogens of soybean worldwide. It is estimated that this disease causes more than one billion dollars in yield losses annually in the United States alone [17]. Conventional management strategies for *H. glycines* including resistant cultivars, crop rotation and nematicides are comprised by limitations [18, 19]. Current resistance cultivars for SCN management, for example, depends heavily on a single resistance source (PI 88788) in cultivar development in the United States, and it displays variable effectiveness across diverse *H. glycines* populations [19, 20]. This has led to concerns about intensive selection for virulence in natural nematode populations [20, 21]. Thus, alternative and novel methods to control this widespread pest are needed to supplement current management strategies. The rapid development of genomics in recent years provides great opportunities for investigating the molecular mechanisms of the interaction between soybean and nematode systems. Increasing evidences reveal that host endogenous small RNAs also play important roles in response to biotic and/or abiotic stress.

Many conserved and legume-specific miRNAs have recently been identified in soybean using high-throughput sequencing and bioinformatic analysis [22–25]. A number of these miRNAs have been shown to be responsive to abiotic stresses, including salinity, drought and chilling stress, phosphate deficiency, and to biotic stresses such as soybean rust and cyst nematode infection [12, 26–30]. Little is known, however, about the function and mechanisms of miRNA regulation during pathogen infection. Here, we identified a set of new small RNAs that are responsive to SCN infection in both susceptible and resistant soybean roots at different developmental stages, and provided a synopsis of the soybean miRNA and SCN interaction system. In total, 60 miRNAs belonging to 25 families were shown to be significantly differentially expressed in response to SCN infection.

## Results

### Soybean inoculation with SCN

**Fig. 1:**
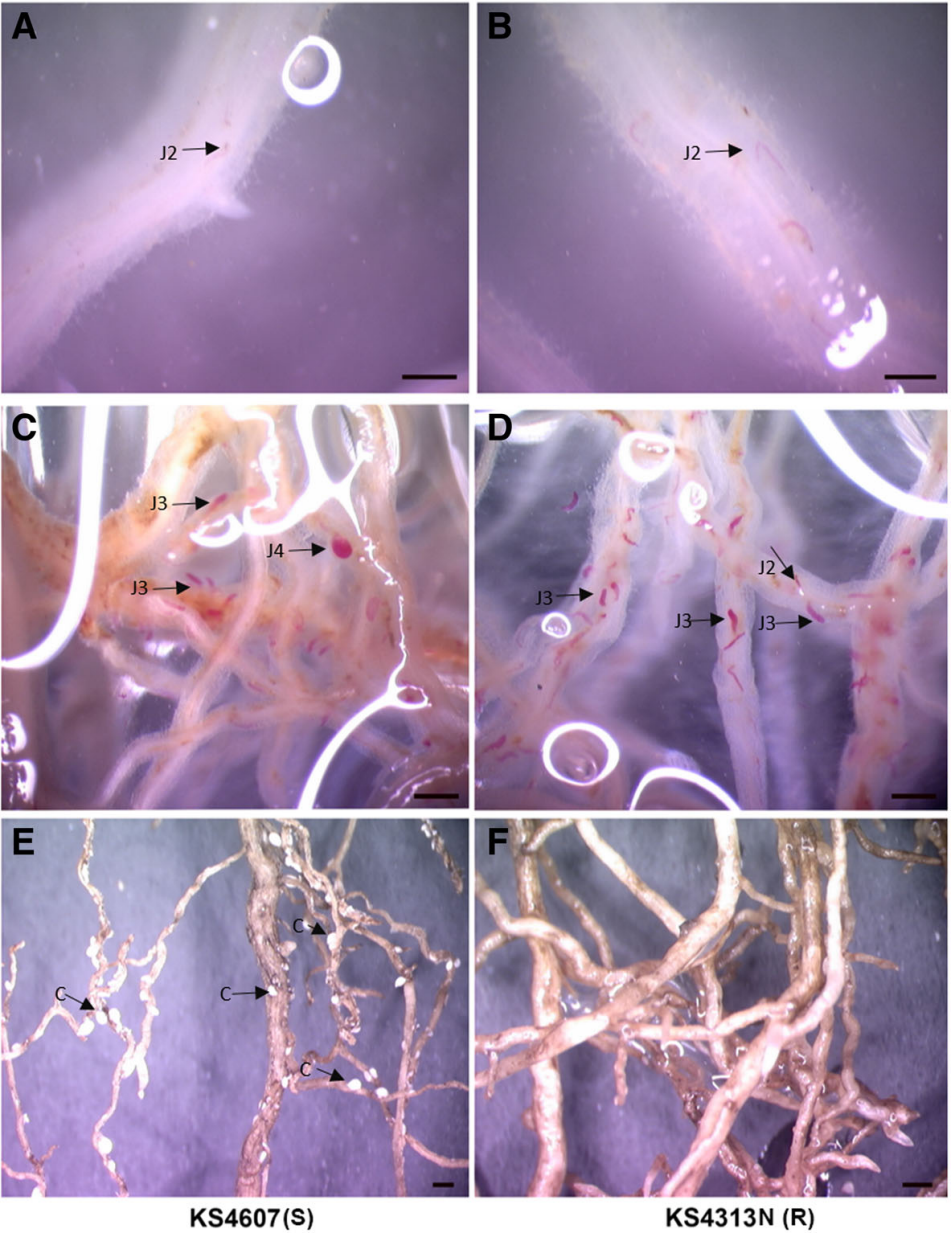
SCN infection at different times. The phenotype of SCN HG Type 7 infected soybean at 4 dpe on a susceptible (*s*) cultivar KS4607 (**a**) and a resistant (*r*) cultivar KS4313N (**b**); SCN J3, J4 and cysts were presented in roots at 30 dpe on KS4607 (**c**) and KS4313N (**d**); The soybean roots infected with SCN were observed at 30 dpe on KS4607 (**e**) and KS4313N (**f**); SCN cysts were observed on susceptible cultivar (**e**). The scale bar represents 1 mm

To understand the role of soybean miRNAs in response to SCN infection, an experiment was conducted with soybean grown in sterilized soil and soil infested with SCN HG type 7. Two Kansas soybean cultivars were used: KS4607, a SCN susceptible cultivar [31], and KS4313N, a cultivar resistant to HG type 7 [32]. Two time points were selected for monitoring miRNA abundance: juvenile establishment at 7 days post emergence (dpe), and cyst production at 35 dpe. Previous studies [33–35] showed that soybean gene expression could be initiated in response to the establishment of SCN infection as early as three days after inoculation. Preliminary assays were carried out to determine the optimal time point at the establishment stage of SCN infection, where soybean seedlings were examined every day for ten days after germination. As expected, a number of second-stage juveniles (J2) of SCN could be observed in the roots of both cultivars after 4 dpe (Fig. 1a and b). Soybean plants were also examined at 35 dpe (Fig. 1c-f) as the SCN life cycle is approximately 30 days after feeding site establishment. In the soybean resistant cultivar KS4313N, although many J2 and J3 nematodes were observed in the root after staining (Fig. 1d), very few cysts were observed on the roots (Fig. 1f). In contrast, the SCN appeared to complete their life cycle during this time in susceptible cultivar KS4607 as many cysts were produced and were visible on roots (Fig. 1e), while less numbers of juveniles were observed in the roots (Fig. 1c). Based on these observations collection of small RNA samples from root tissue were subsequently performed at 7 dpe and 35 dpe.

### Computational identification of miRNA and sRNA from small RNA-seq libraries

**Fig. 2:**
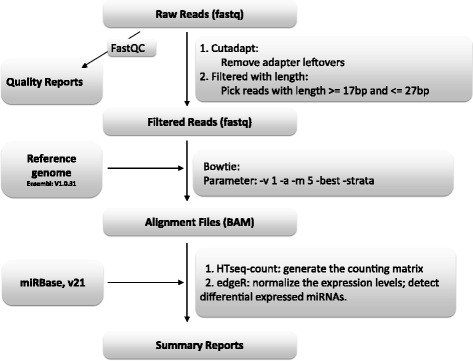
Analyses workflow of sRNA libraries. Pipeline used to identify soybean sRNAs regulated in response to SCN infection

**Fig. 3:**
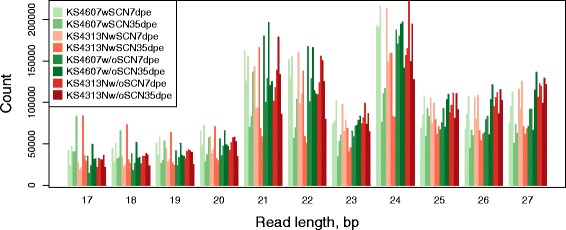
Sequence length distribution of small RNA in different libraries of soybean roots

Small-RNA libraries prepared from SCN-free plants and plants inoculated with SCN were sequenced by Illumina technology. Small-RNA libraries were constructed for each of three biological replicates of the eight experimental treatments (SCN infested vs, non-infested, and two time points, for two soybean genotypes). These 24 soybean root small RNA libraries provide a total of 323,433,859 sRNA raw reads. The bioinformatics pipeline used in the sRNA analysis was shown in Fig. 2. After low quality and adapter sequences were removed, 58,628,603 reads ranging from 17 to 28 nucleotides (nt) were used for further analyses. As shown in Fig. 3, the highest abundance was found for sequences with 21, 22 and 24 nucleotides (nt), which is consistent with previous studies in soybean small RNA sequencing analyses [12, 23, 36]. There was no obvious difference in the length distribution among the two cultivars and SCN infection libraries. Small RNAs in 24-nt class showed higher abundance to 21-nt and 22-nt. Using Bowtie v1.2.0 [37] to align small RNA reads, allowing non-mismatch, against the soybean genome *Glycine max* v109 [38] in the Phytozome database version 12.0 [39], a total of 7,642,558 and 6,706,663 small RNA sequences were mapped in the SCN-free and SCN-inoculated libraries, respectively. From these mapped reads, a search in the miRBase database (release 21) [40], identified 225 and 251 known miRNAs with at least 5X coverage in control libraries for KS4313N and KS4607 cultivars, respectively; Additionally, 231 and 237 known miRNAs had been identified in SCN infected libraries for KS4313N and KS4607 cultivar, respectively (As shown in Additional file 1: Table S1).

### Changes in abundance of known soybean miRNA upon SCN infection

The sequencing frequency, the “counts per million” (CPM), of each of miRNA in each library was used as an index for estimating the relative abundance, the expression level of each sample. The comparison among treatments showed a dynamic miRNA regulation in response to SCN infection. While 90.3% of known miRNA in soybean were identified in both library sets, gma-miR5037c (1520a, 1520 h, 1528, 1529, 167 h, 167i, 169c, 169i, 169p, 4355, 4359a, 4377, 4380a, 4394, 4399, 5032, 5037c, 5780a, 5782, 9739,9740, 9745) were found only in the infestation libraries, and gma-miR1518 (1520p, 1524, 1534, 169 t, 4351, 4352a, 4386, 4406, 5044, 5371, 5672, 5762, 5764, 9744, 9747, 9748, 9754, 9765) were present only in the control libraries. The most abundant family was gma-miR1507ab in all libraries, followed by members of miR166, miR1510, and miR3522. The most abundant miRNAs in all libraries were conserved miRNAs such as miR482, miR159, and miR396, while several legume specific miRNAs, such as miR1510, miR2109, miR2118, miR4996, and miR1509, were also highly abundant. Further analysis demonstrated that conserved miRNAs have relative high abundance in general and without significant difference among infected and un-infection samples, therefore, the results indicated that conserved miRNAs may be critical and essential for fundamentally cellular growth and development in soybean roots. More than two thirds of the miRNAs detected in all libraries had low expression levels, with fewer than average 100 CPM in each library.

**Fig. 4:**
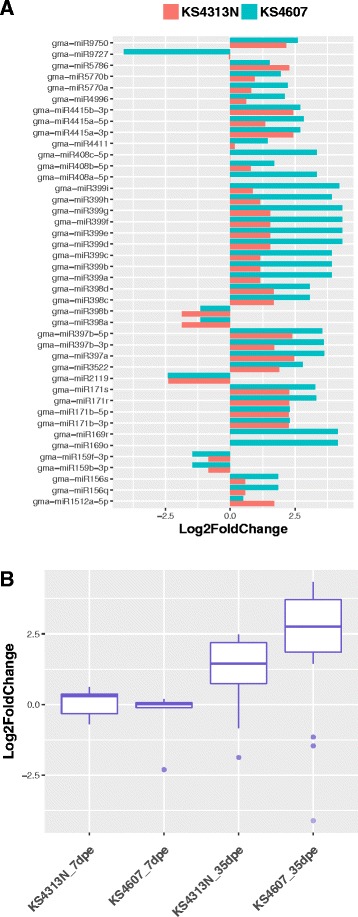
Expression levels of differentially expressed (DE) miRNA in soybean responsive to SCN infection. **a** Comparison of DE miRNAs expressed in two soybean genotypes at 35 dpe. **b** The expression level changes of all DE miRNAs were identified in two genotypes at different time points

To identify SCN infection responsive miRNAs in soybean roots, miRNA expression profiling was compared between two time points SCN infested and corresponding control libraries in both genotypes. Using 0.05 as the q-value (adjusted *p*-value) threshold for miRNA expression difference, a total of 60 miRNAs belong to 25 families were found to be differentially expressed (DE) from at least one treatment (Table 1 and Additional file 2: Table S2). In the early stage of SCN infection (i.e. 7 dpe), only two miRNAs, miR398a and miR398b, were significantly down regulated in KS4607, while 18 miRNAs were either up-regulated or down-regulated in KS4313N with all fold changes (FC) < 2 (Table 1 and Fig. 4b). Most DE miRNA were identified in the late stage at 35 dpe. The largest number (34) of DE miRNAs belonging to 15 families were up-regulated during the SCN infection in susceptible cultivar KS4607, while only three miRNAs (miR159b-3p, miR159f-3p, and miR9727) were down-regulated (Fig. 4a). The resistant cultivar KS4313N showed a similar trend, with 14 miRNAs belonging to 9 families displaying up-regulated, and only miR2119, miR398a and miR398b displaying down-regulated (Fig. 4a). In general, all DE miRNAs identified at 35 dpe had stronger responses in KS4607 compared to the resistant cultivar KS4313N (Fig. 4b). Among DE miRNAs responsive to SCN infection, two miRNAs (gma-miR2119, and gma-miR1512a-5p) were found to be unique in the resistant cultivar KS4313N, while 9 miRNA families (gma-miR399, 169, 156, 159, 408, 4411, 4996, 5770, and 9727) were significantly differentially expressed in only KS4607 but not in KS4313N. Additionally there were 8 DE miRNA families common in both cultivars at 35 dpe (Additional file 2: Table S2).

**Table 1:** Summary of differential expression of miRNAs in soybean roots

Comparison to control inoculations	Significant miRNAs	Up-regulated		Down-regulated	
		All	> = 4 FC	All	> = 4 FC
KS4607 (S) at 7dpe	2	0	0	2	2
KS4607 (S) at 35dpe	37	34	29	3	1
KS4313N (R) at 7dpe	18	13	0	5	0
KS4313N (R) at 35dpe	17	14	10	3	1

(S) – SCN HG type 7 susceptible soybean cultivar; (R) – SCN HG type 7 resistant soybean cultivar

**Fig. 5:**
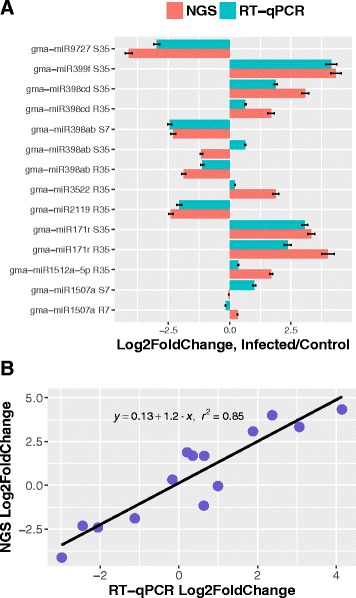
Selected miRNAs were experimentally tested by RT-qPCR confirming expression patterns of deep sequencing. **a** Most miRNAs were consistent with the expression patterns of deep sequencing; **b** Regression analysis between NGS and RT-qPCR indicated a strong correlation with R^2^ = 0.85

To confirm the sequencing results, we also performed experimental RT-qPCR using the same RNA samples to validate several miRNAs that were represented as conserved and soybean specific miRNAs with differentially expressed in at least one treatment. Most miRNAs (12 out of 14) were consistent with the sequencing data although fold changes displayed varying degrees of divergence in most cases (Fig. 5a). For example, gma-miR398a and gma-miR398b were down-regulated with log_2_ ratio − 1.15 in susceptible cultivar at 35 dpe in our sequencing results, while they were up-regulation with the log_2_ ratio 0.63 in RT-qPCR experiments. The regression analysis between two approaches was indicated a relative high correlation as R^2^ = 0.85 (Fig. 5b). This was likely due to sequence bias introduced by small RNA libraries or profiling miRNA RT-qPCR, or to different normalization approaches employed in these two strategies.

### Identification of novel soybean miRNA

The search for novel miRNA candidates was conducted using the miRDeep2 pipeline mapped to *G. max* genome v.2 with a blast-n search against the miRbase database, release 21 [40]. We applied the miRDeep2 module to analyze the RNA-seq reads for novel miRNA prediction because the miRDeep2 algorithm has been widely used for miRNA precursors identification due to its accuracy and sensitivity [41, 42]. Those identified the precursors were detected at least in two libraries with a miRDeep2 score of 1 or greater. Hence, 70 novel gma-miRNA candidates were discovered as shown in Additional file 3: Table S3.

### Target function prediction of soybean miRNA

**Fig. 6:**
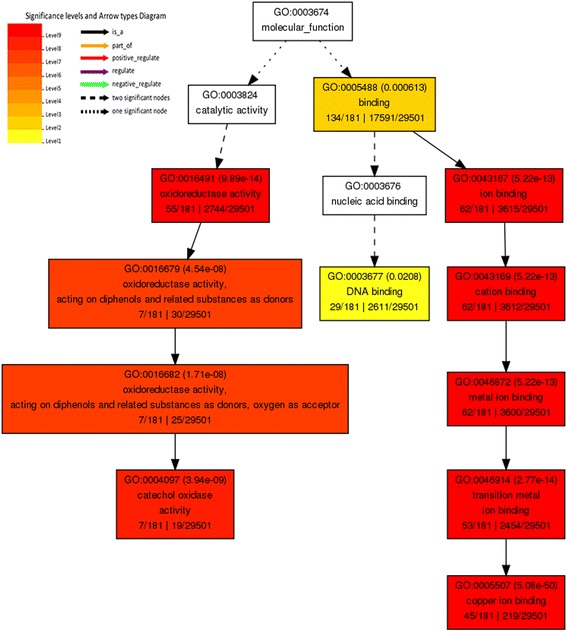
Gene ontology (GO) enrichment analyses. It was performed for all miRNA target genes in soybean roots, containing only the predicted target genes for differentially expressed miRNAs. The common GO were shown here, and plant oxidative response was the most related biological process to SCN infection. The output is a GO hierarchical image including all statistically significant terms. Red color means a higher statistical significance of terms

**Fig. 7:**
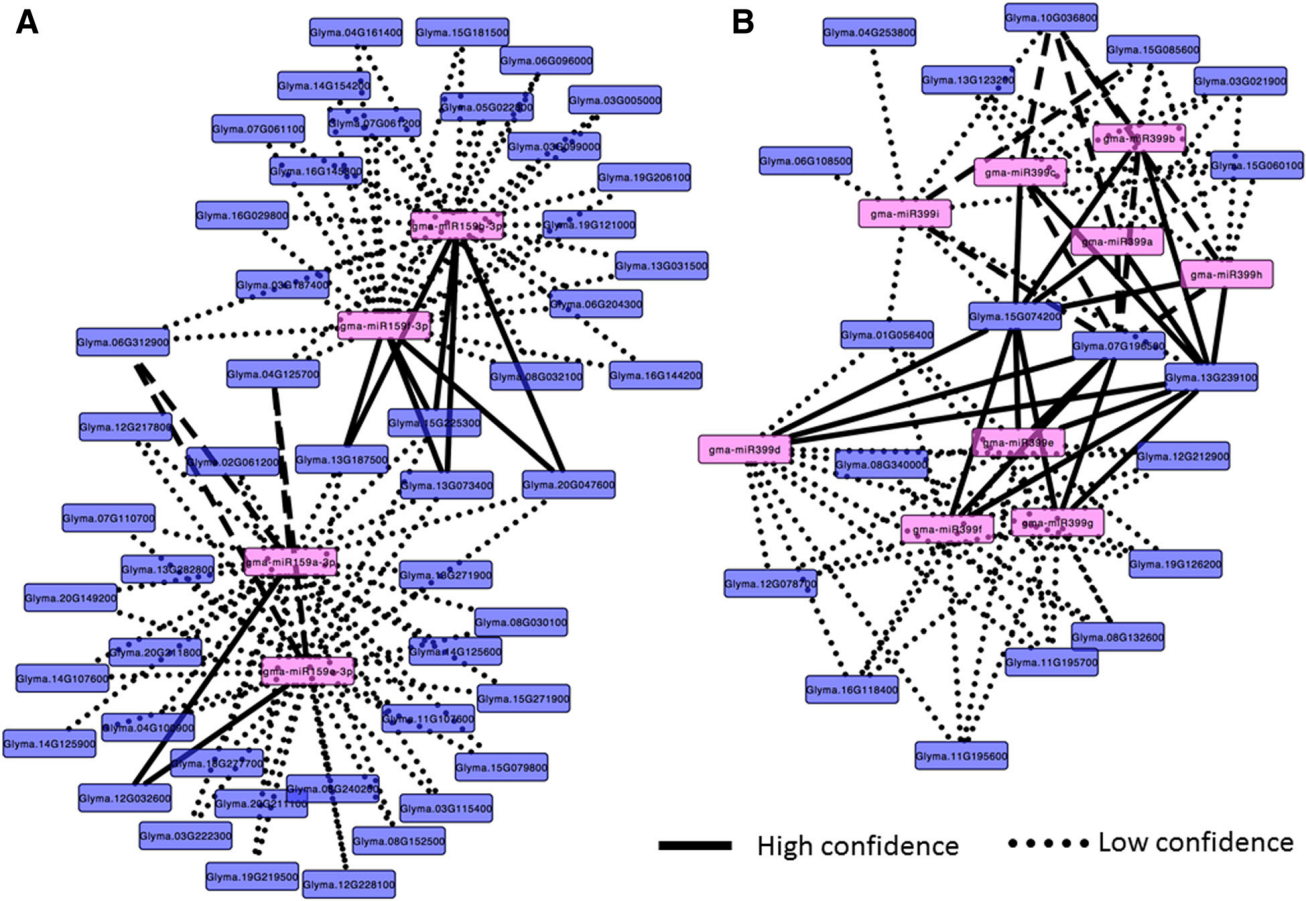
The regulation network between two SCN induced miRNAs and their targets in roots. **a** miR159 family and their targets responsive to SCN infection; **b** miR399 family and their targets responsive to SCN infection

To understand the biological mechanisms triggered by miRNAs in response to SCN infection of soybean roots, we conducted gene ontology (GO) analysis of the regulated miRNAs’ target genes as shown in Additional file 4: Table S4, which were identified by psRNATarget [43]. The gene ontology of these targets then was constructed by agriGO [44]. The results demonstrated that the target genes of identified DE miRNAs are mainly classified into three molecular functions including oxidative activity, ion binding, and nucleic acid binding (Fig. 6). The oxidative reaction is the major biological process, and it may be related to the first defense to SCN. Among the DE miRNAs, several members in conserved miR159 and miR399 families were significantly changed in both cultivars. To understand and visualize the regulation network between these two families and their targets, regulatory network analysis was performed as shown in Fig. 7. MiR399 had strong connection with a number of different targets. The regulatory network revealed that miR159 and miR399 likely had a function in response to SCN infection by targeting a number of different genes in roots.

## Discussion

In the present study, we sequenced small RNA libraries from the roots of two soybean genotypes at different time points in the presence and absence of SCN infection and acquired 0.3 billion reads. The large data set allowed us to identify miRNA with low abundance, and reduced the experimental variation. In total, 574 known miRNAs and 70 novel miRNAs were identified from soybean roots, representing about 90% of known miRNAs in the current miRBase (release 21).

Although miRNAs have been implicated in plant-microbe interactions, questions remain regarding how many miRNAs are involved and whether pathogenic or mutualistic interactions share common sets or employ distinct sets of miRNAs as components in their interacting signaling pathways. In our study, a larger dataset with biological replicates and controls were applied to provide reliable analysis to identify miRNAs that are closely associated with pathogen interaction.

We reasoned that the identification of DE miRNAs during the SCN infection would result in better understanding of the plant endogenous regulation in soybean during SCN infection. We compared the expression level of miRNAs between SCN-infected and SCN-free samples in two cultivars at two time points, and identified a total of 60 differentially expressed miRNAs as SCN responsive. Most of DE miRNAs were induced at 35 dpe, and were up-regulated in infected roots. Similar phenomenon have been observed for cyst nematode infection in Arabidopsis, where the majority of significantly DE miRNAs were also up-regulated, especially late in the infection process [45]. At the early stage of SCN infection, only two DE miRNAs (gma-miR398a and gma-miR398b) were observed in susceptible KS4607, while the fold changes of 8 DE miRNAs families in resistant cultivar KS4313N were small (FC < 2), suggesting the general effects of plant miRNA regulation on SCN root penetration seems not momentous. It may be explained that there was no significant difference observed for SCN juveniles invading between susceptible and PI88188 based resistant cultivars (Fig. 1) in our study. Further, there are several miRNA families both significantly regulated in both libraries of two soybean genotypes, including several conserved miRNAs such as gma-miR397, 398, and 171, and legume-specific miRNAs, gma-miR3522, 4415, 5786, and 9750. Although little are known for the functions of some miRNA families, these miRNAs may play a general role in developmental and/or defensive responses to pathogenic stresses.

**Fig. 8:**
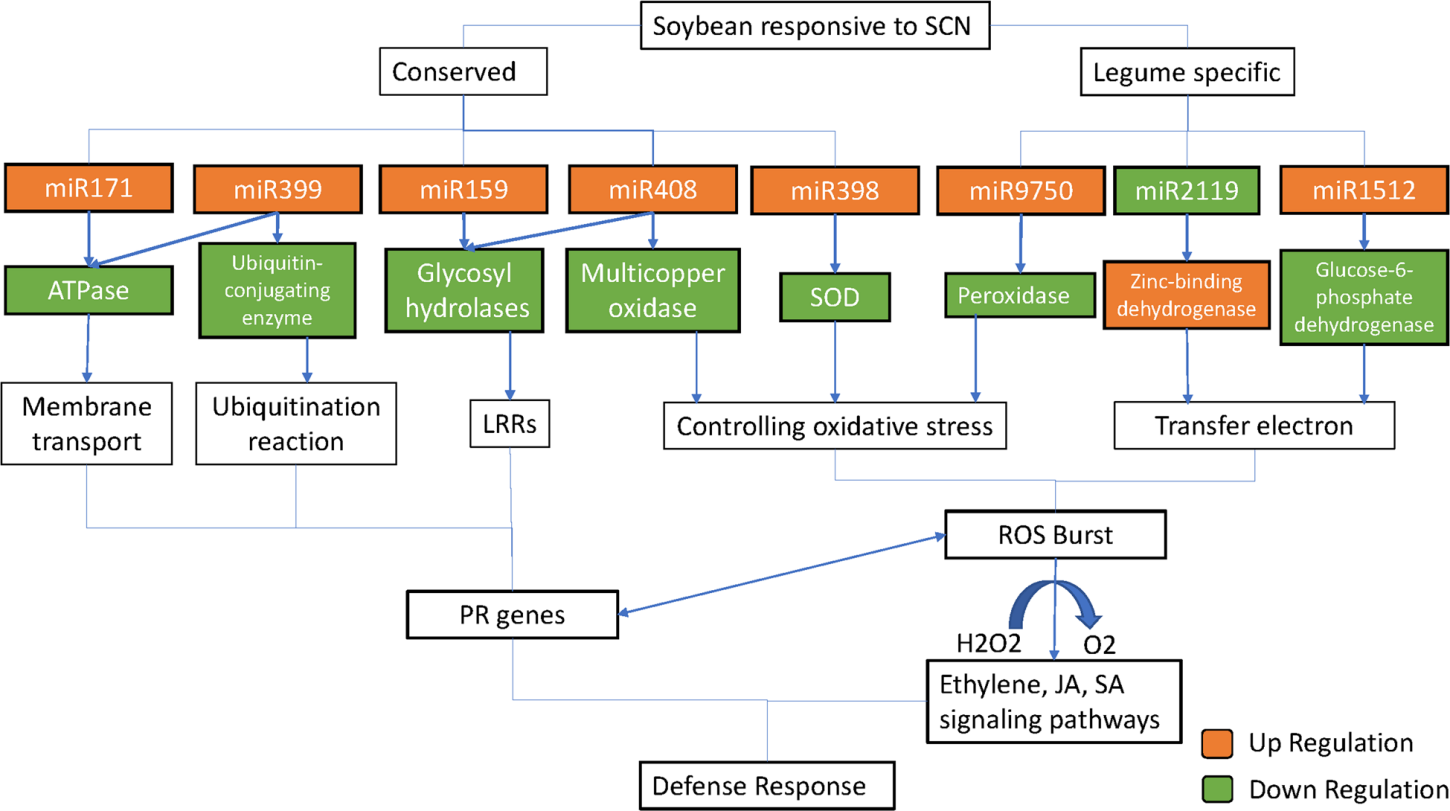
Potential regulatory mechanism of DE miRNAs and their targets involved in the soybean and SCN interaction

The miRNA-involved regulation of plant-pathogen interactions mainly depends on the relative factors of plant immunity and pathogen infection. Direct pathogen infection could immediately trigger and activate the plant host immunity response. It may require miRNAs acting as key regulators of R genes and/or plant hormones. Expression of gma-miR399 and gma-miR408 were significantly induced by SCN in the susceptible cultivar KS4607 only. Expression of miR399 has also been reported to be specifically induced by infection of *Candidatus L. asiaticus*, the bacterial causal agent of citrus greening disease [46]. Another conserved miRNA, miR159, was revealed to be induced by *Pseudomonas syringae* infection to regulate the ABA and/or gibberellin signaling pathway [47]. By comparison, miR408 was shown in wheat and Arabidopsis to target plantacyanin-like proteins, which play an important role in wheat resistance to stripe rust [48]. Furthermore, miR169 and miR171, which are involved in powdery mildew infection in wheat [49], were also significantly induced by SCN in both susceptible and resistant cultivars (Additional file 2: Table S2). Specially, two unique DE miRNAs, gma-miR2119 and gma-miR1512a, only in the resistant cultivar KS4313N were down-regulated and up-regulated, respectively, suggesting these conserved legume miRNAs may have regulation function specific to SCN resistance. Similarly, gma-miR2119 was also reported as one of the highest alternation miRNAs down-regulated by SCN race 3 infection in a resistant soybean cultivar HB [12]. For gma-miR1512, it has been reported to be associated to rhizobial symbiosis [50]. With GO enriched analyses of their targets, putative functions were derived from the SoyBase annotation (http://www.soybase.org) [51]. Both miRNAs were likely regulating different dehydogenases (Fig. 8) in responsive to pathogen reaction. A suppression of Glucose-6-phosphate dehydrogenase (G6PD) isoform was approved to enhance plant stress tolerance in general [52]. While, the induced alcohol dehydrogenase (ADH) have been reported in the interaction between the resistant plants and pathogens [53, 54], including ADH upregulation in the incompatible interactions among the cyst nematode and tomato plants [55]. More recently, an overexpression of the peanut zinc-binding dehydrogenase 2 (adZADH2) was also reported to enhance the resistance to late leaf spot pathogens [56].

In response to plant immunity, pathogens have evolved effectors to interfere with host defense responses, facilitating infection. Many effectors have been identified to interfere with host miRNAs to promote pathogen infection [15, 16, 57]. At the initial infection stage for the susceptible cultivar KS4607, significant changes in abundance were observed only for miR398a and miR398b. In contrast, more miRNAs were responsive to SCN infection in the resistant cultivar KS4313N, but with relatively minor fold changes (Fig. 4b). One of most abundant legume specific miRNAs in soybean, miR1507, was induced at an early stage but only in the resistant cultivar. This miRNA has been determined to target conserved domains in NBS-LRRs and trigger the production of phasiRNAs in *Medicago truncatula* [58]. Similarly, miR482 in tomato is only responsive to *Fusarium oxysporum* infection in resistant cultivars [59], and miR393, which is involved in the auxin immunity pathway through inhibition of auxin receptors, is only responsive in resistant Arabidopsis plant too [10]. Three differentially expressed conserved miRNAs (gma-miR393, gma-miR398 and gma-miR399) have also been reported previously to be promising varied in two sister soybean lines that are resistance and susceptible to SCN race 4 [27]. All evidence was suggesting that the candidate miRNAs were likely involved in SCN infection process in soybean.

It is notable that members of the miR398 family had opposite expression patterns at different infection stages in our study. MiR398 was shown to be involved in regulating the rapid accumulation of reactive oxygen species (ROS), which is responsive to diverse biotic and abiotic stress in plants [60, 61]. It is known that the down-regulation of miR398 in Arabdopsis results in releasing its suppression of Superoxide dismutase 1 (CSD1) and Superoxide dismutase 2 (CSD2) genes that plants could improve the tolerance to the oxidative stress [60], which could be the first line of plant defense [62]. During pathogenesis, the unchanged and/or up-regulated miR398, and consequently, the repression of CSDs, may suggest plant defense responses were not trigger by some beneficial endophytes such as *Herbaspirillum seropedicae* and *Azospirillum brasilense* in maize [63]. It is possible that miR398 may also play dual roles in response to SCN infection, as gma-miR398a and gma-miR398b were significantly down-regulated at 7 dpe but no significant changes at 35 dpe in the susceptible cultivar KS4607, whereas, they were down-regulated at 35 dpe in the resistance cultivar KS4313N. Meanwhile, miR398c and miR398d were both up-regulated in both susceptible and resistant cultivars at 35 dpe. Although miR398 members were mostly related to abiotic stress, it has been also reported to be responsive to various pathogen infections. In *Arabidopsis*, miR398b was down-regulated during flg22 treatment [64], and it was also involved in the basal response of rice to the blast fungus *Magnaporthe oryzae* [65]. This was consistent with the observation that gma-miR398a and gma-miR398b were both down-regulated during the initial stage of infection on KS4607 and the later stage of infection on KS4313N. Further insight into the role of miR398 comes from the observation that miR398 plays a crucial dual but opposite roles during normal growth and development process and abiotic or biotic stresses [61]. This family appears to be one strong potential candidate for miRNAs involved in soybean responses to SCN infection.

To date, a number of studies have been designed to discover miRNA responsive to various abiotic and biotic stresses. In our study, most of differentially expressed miRNAs were up-regulated by SCN infection, in contrast to a previous report by Li et al. (2012) that the majority of SCN-responsive miRNAs were down-regulated by infection. This discrepancy could be related to different soybean cultivars used in these experiments, and/or the different experimental designs and analyses employed. Unfortunately, lack of biological replicates in the previous report [12] might result in strong bias on differential expression analysis. Here, we conducted three biological replicates for each treatment. From expression analysis among replications (Additional file 5: Figure S1), we could see differential expression existing among biological replicates, indicating that replications are essential for differential gene expression analysis in RNA-seq experiments. Recent reports [66, 67] also analyzed and emphasized the importance of experimental replicates for providing sufficiently comprehensive view of the differential expression analysis to inform the biology meaning accurately. It could be even especially critical for analyzing complicated environments, such as plant responses to soil-borne pathogens. In any event, the discrepancy needs to be further investigated.

Recently developed short tandem target mimic (STTM) technology, which contains two miRNA binding modules plus an empirically tested linker of 48–88 nt, inducing the degradation of targeting endogenous small RNAs partly through the small degrading nucleases in plants have been successfully applied to demonstrate functions of many miRNAs [68–70]. STTM provides a highly effective approach that can specifically degrade and suppress all members in one miRNA family simultaneously, and consequentially up-regulate corresponding target genes. This technology has been applied to knocking down miRNA in major crop plants including soybean, rice, and tomato [71, 72]. Furthermore, soybean transgenic roots with STTM-reduced abundance of miR393 resulted in significant susceptibility to *Phytophthora sojae* infection [73]. With several important miRNAs identified and possible regulatory mechanism hypothesized in this study (Fig. 8), STTM could be a useful tool for developing new strategies to improve soybean resistance to SCN.

## Conclusions

Soybean is an important protein and oil crop. However, the management for SCN problems, the most severe disease of soybean worldwide, was very limited to a few resistant sources. It is beneficial to explore the new areas for this pest control. Based on the comparative small RNA-seq analyses of miRNAs in soybean roots responsive to SCN, it indicated that soybean plants may use a variety of miRNAs regulation mechanisms to response and regulate this plant-pathogen relationship. In conclusion, a total of 60 DE miRNAs were identified related to SCN responses using multiple replicated treatments. According to the functional analysis and previous reports, several conserved miRNAs (for example, gma-miR159, gma-miR171, gma-miR398, gma-miR399, and gma-miR408) and legume specific miRNAs (for example, gma-miR1512, gma-miR2119, and gma-miR9750) could be potential candidates for manipulating the SCN infection. These findings will provide a framework and new sight for small RNA engineering approach to fight SCN pathogenic infection.

## Methods

### Plant material and SCN inoculation

Two soybean genotypes ‘KS4607’ and ‘KS4313N’, which are susceptible and resistant to SCN HG type 7, respectively, were used in this study. All seeds were surface sterilized before germination as described by Tian et al. [74]. Healthy germinated seeds were planted into brand new D40 Deepots (Stuewe and Sons, Inc., Corvallis, OR) with sterilized soil and SCN-infested sterilized soil containing approximately 3000 eggs per 100 cm^3^ of an HG type 7 *H. glycines* population. The SCN population originated from a naturally infected soybean field in Cherokee Co., KS and was maintained in the greenhouse on susceptible soybean cultivar KS3406. The plants were grown in a growth chamber under 26 °C/24 °C and 16/8 h light/dark photoperiod, with the experimental treatments arranged in a completed randomized block design.

### Sample preparation and RNA isolation

Three independent biological replicates per treatment were grown under the same conditions as described above, and soybean roots were collected seven days and 35 days post inoculation. Roots were cleaned up with high pressure tap water as standard SCN bioassay procedure as described by Tian et al. [74], and flash frozen in liquid nitrogen and then stored at −80 °C until RNA extraction. Treatments were as follow: KS4607 non-inoculated at 7 dpe (S7C), KS4607 non-inoculated at 35 dpe (S35C), KS4607 SCN inoculated at 7 dpe (S7 N), KS4607 SCN inoculated at 35 dpe (S35 N), KS4313N non-inoculated at 7 dpe (R7C), KS4313N non-inoculated at 35 dpe (R35C), KS4313N SCN inoculated at 7 dpe (R7N), and KS4313N SCN inoculated at 35 dpe (R35N). Three plants for each sample were pooled for RNA extraction. Total RNA from each replicate was extracted separately with TRIzol reagent (Invitrogen, Carlsbad, CA, USA) according to the manufacturer’s instructions. To improve the quality and quantity of total RNA, a 2 mL Phase Lock Gel Heavy (5PRIME GmbH, Hilden, Germany) was used according to the manufacturer’s instruction. The total RNA quantity and purity were analysis by Tape Station and Bioanalyzer 2100 (Agilent, CA, USA).

### Small RNA libraries construction and deep sequencing

The small RNA libraries were constructed using the TruSeq Small RNA Sample Preparation kit according to the manufacturer’s instructions. The single-end 50 cycle sequencing was performed using Illumina HiSeq2500 platform at Genome Sequencing Core at University of Kansas (Lawrence, KS), which produced 50 single-end reads.

### Differential expression analysis of miRNAs

Raw sequencing reads were first evaluated using FastQC [75], then trimmed to remove the low quality bases and adapter leftovers using cutadapt [76]. After trimming, reads ranging from 17 to 27 nt were collected for subsequent analysis. The processed reads were aligned to soybean reference genome (plant ensemble, v1.0.31) [38] using bowtie v1.2.0 [37]. The uniquely aligned reads were then run through miRDeep2 module for quantifying known miRNAs (mirBase V21) as well as discover novel miRNAs. The raw read count matrix for miRNAs were supplied to edgeR [77] for detect differential expressed miRNAs among groups. Briefly, the raw read count data was first scaled to library size followed by normalizing with weighted trimmed mean of the log expression ratios (also known as trimmed mean of M values, TMM) to consider the compositional bias in sequenced libraries; then the dispersion of the reads counts was estimated and an exact test was performed to detect differential expressed miRNAs between the groups. We applied the miRDeep2 algorithm to analyze potential novel miRNAs expressed in the samples. The novel gma-miRNA candidates were identified if detected at least in two libraries with a prediction score of 1 or greater, as suggested by the authors of miRDeep2.

The potential targets of miRNA were predicted using the psRNATarget server (http://plantgrn.noble.org/psRNATarget/) by providing the sequences of all small RNAs in the mirBase for soybean. To identify the enriched GO groups in the differentially expressed miRNAs, the predicted targets for those miRNAs were supplied to the online GO analysis server AgriGO (http://bioinfo.cau.edu.cn/agriGO/) while using the predicted targets of all expressed miRNAs in our samples as reference/background. Go terms that have *p*-value <= 0.05 are chosen as GOs enriched in the examined target gene set. The targets in the regulatory network were also predicted using the online tool psTNATarget with high confidence (Expectation <1) and low confidence (Expectation >3). The network was visualized using Cytoscape v3.4.0 (http://www.cytoscape.org/).

### Quantitative RT-PCR analysis for miRNA expression

To validate the expression of identified miRNAs in our RNA-seq analysis, several miRNAs with different expression patterns in different treatments were randomly selected for RT-qPCR. The Mir-XTM miRNA First-Strand Synthesis Kit (Clontech, Mountain View, CA) was used for polyadenylation and reverse transcription of all miRNAs at one time according to the manufacturer’s protocol. qPCR were performed on the ABI 7900HT Fast Real-Time PCR system (Applied Biosystems, USA) using Mir-X™ miRNA qRT-PCR SYBR® Kit (Clontech, Mountain View, CA). The qPCR program was set up with one cycle for template denaturation and hot start Taq activation at 95 °C for 2 min, then 40 cycles of 95 °C for 5 s, and 60 °C for 20 s extension step with dissociation. The conserved miR171a was used as an internal reference gene for normalization in soybean root samples [78]. The RT-qPCR reaction was performed according the manufacturer’s protocol with three biological and three technical replicates for each sample. In the expression analysis, all values were analyzed using the 2^–∆∆CT^ method [79] and were standardized to relative log_2_ fold changes for all inoculated and control samples. All values were presented as means with standard deviation (SD).

## Additional files

Additional file 1: Table S1. Total known miRNAs identified in *Glycines max* from all small RNA-seq libraries. The value was the mean of three replicates for each treatment and normalized as CPM. (XLSX 51 kb)

Additional file 2: Table S2. Significantly Differentially expressed miRNAs identified in SCN-infected soybean roots compared with control. A table of all DE miRNAs identified from each treatment. The *q*-value was used to consider for statistical significance, with log_2_ ratio of normalized miRNA expression in SCN infection compared with control. (XLSX 14 kb)

Additional file 3: Table S3. Novel miRNA prediction. A table of new miRNAs predicted by miRDeep2 with a score of one or greater. (XLSX 21 kb)

Additional file 4: Table S4. Target prediction of DE gma-miRNAs identified. A table of all predicted targets of DE miRNAs using psRNATarget online. (XLSX 46 kb)

Additional file 5: Figure S1. Expression levels of all RNA-seq libraries. The boxplot of CPM normalized expression for all 24 libraries. (PDF 6 kb)

## Abbreviations

ADH: alcohol dehydrogenase
CPM: counts per million
CSD: Superoxide Dismutase
Ct: Cycle threshold
DE: Differentially Expressed
dpe: days post emergence
FC: Fold Changes
G6PD: Glucose-6-phosphate dehydrogenase
GO: Gene Ontology
J2: second-stage juveniles
miRNA: microRNA
NBS-LRR: Nucleotide-binding Site Leucine Rich Repeat
NGS: next generation sequencing
nt: nucleotides
ROS: Reactive Oxygen Species
SCN: Soybean Cyst Nematode
SD: Standard Deviation
STTM: Short Tandem Target Mimic
ZADH: zinc-binding dehydrogenase
