# Supplementary figures and images for: Genome-wide identification of soybean microRNA responsive to soybean cyst nematodes infection by deep sequencing
Source: BMC Genomics. 2017 Aug 2;18:572. doi: 10.1186/s12864-017-3963-4 (PMC5541722; doi:10.1186/s12864-017-3963-4)

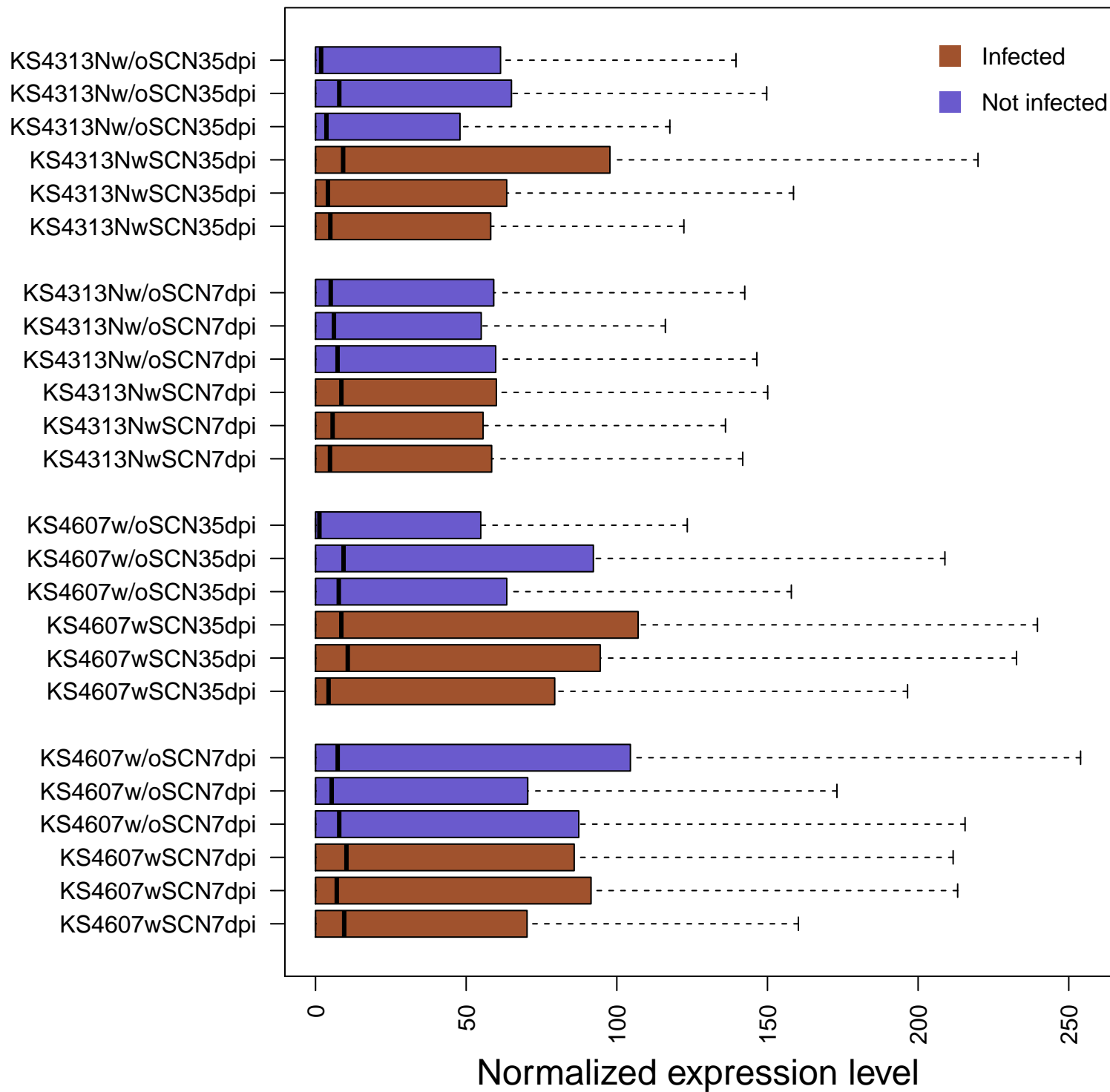

Supplement: Supplementary file 5 — Expression levels of all RNA-seq libraries. The boxplot of CPM normalized expression for all 24 libraries. (PDF 6 kb) [file 12864_2017_3963_MOESM5_ESM.pdf]
